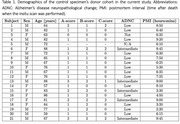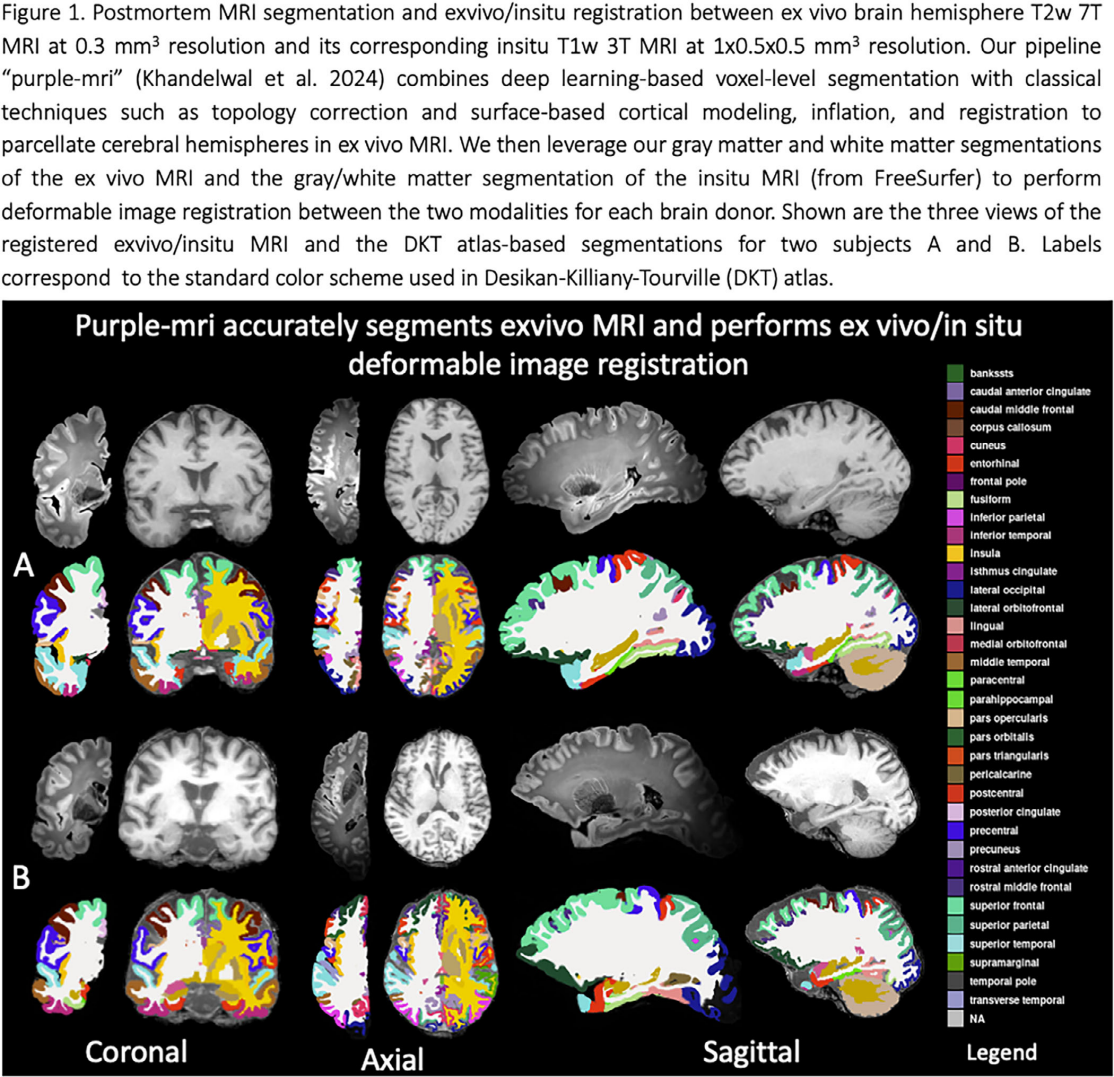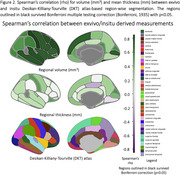# Segmentation and registration of within‐subject 7 tesla ex vivo and 3 tesla in situ MRI to increase sensitivity of imaging biomarkers

**DOI:** 10.1002/alz70856_101680

**Published:** 2025-12-25

**Authors:** Pulkit Khandelwal, Sydney A. Lim, Wilma D.J. Van de Berg, Louise van der Weerd, Paul A. Yushkevich, Laura E. Jonkman, Normal Aging Brain Collection Amsterdam

**Affiliations:** ^1^ Penn Image Computing and Science Laboratory (PICSL), University of Pennsylvania, Philadelphia, PA, USA; ^2^ University of Pennsylvania, Philadelphia, PA, USA; ^3^ Department of Anatomy and Neurosciences, Amsterdam UMC, Vrije Universiteit Amsterdam, Amsterdam Neuroscience, Amsterdam, Netherlands; ^4^ Amsterdam Neuroscience, program Neurodegeneration and Brain Imaging, Amsterdam, Netherlands; ^5^ Leiden UMC, Leiden, Zuid‐Holland, Netherlands

## Abstract

**Background:**

Understanding pathological contribution in mixed etiology dementia and atypical Alzheimer's disease remains challenging. However, in‐vivo MRI and neuropathology studies are hampered by the years of delay between MRI and brain autopsy. Postmortem in‐situ MRI is a good proxy to mitigate this problem (Frigerio et al. 2021) but is logistically challenging. Ex‐vivo MRI offers significant higher anatomical resolution but is influenced by fixation and possible deformation. Therefore, we propose a whole‐hemisphere ex‐vivo to in‐situ/in‐vivo parcellation and registration pipeline “purple‐mri” (Khandelwal et al. 2024) to cater multi‐resolution, translation, and clinical relevance for T2w 7T 0.3 mm^3^ exvivo and T1w 3T 1x0.5x0.5 mm^3^ insitu MRI in 21 (age: 57‐88 years; F=9/M=12) control specimens (Table 1).

**Method:**

We combine a deep learning‐based segmentation architecture with classical surface‐based modeling techniques for topology correction, cortical modeling, inflation, and registration to provide accurate anatomical parcellations of T2w 7T 0.3 mm^3^ exvivo cerebral hemispheres using the DKT atlas. Next, we develop a two‐stage inter‐modality diffeomorphic image registration method between corresponding pairs of exvivo hemispheres and insitu whole‐brain MRI that first aligns segmentations of the two modalities and then applies the obtained warp to perform intensity‐based MRI alignment. FreeSurfer was used to provide DKT atlas‐based segmentations for insitu MRI.

**Result:**

Figure 1 shows qualitative parcellations of exvivo hemisphere and corresponding registered in‐situ MRI. Our method can segment brain regions involving imaging artifacts with signal dropout in anterior/posterior areas. Figure 2 shows Spearman's correlation for region‐wise volume and mean thickness between exvivo and insitu MRI. We observe significant correlations between the two modalities in inferior/middle/superior temporal, insula, lateralorbitofrontal, post/pre central regions for volume‐based correlation. For mean thickness, we observe strong correlation in cuneus, fusiform, medial/lateral orbitofrontal, postcentral gyrus but an overall slight decrease in correlation strength primarily due to noisy thickness measurements due to lack of segmentation in areas with damaged/missing tissues during physical handling.

**Conclusion:**

Our proposed pipeline (purple‐mri) accurately segments high‐resolution exvivo MRI and performs registration to give one‐to‐one correspondence between multi‐modal postmortem MRI. The developed pipeline sets the stage for exvivo to invivo registrations, which would enable stronger association studies between morphometry and histopathological examination.